# Greater Activity in the Frontal Cortex on Left Curves: A Vector-Based fNIRS Study of Left and Right Curve Driving

**DOI:** 10.1371/journal.pone.0127594

**Published:** 2015-05-19

**Authors:** Noriyuki Oka, Kayoko Yoshino, Kouji Yamamoto, Hideki Takahashi, Shuguang Li, Toshiyuki Sugimachi, Kimihiko Nakano, Yoshihiro Suda, Toshinori Kato

**Affiliations:** 1 Department of Brain Environmental Research, KatoBrain Co., Ltd., Tokyo, Japan; 2 Department of Environment/Engineering, Tokyo Branch, Central Nippon Expressway Co., Ltd, Tokyo, Japan; 3 Department of Environment/Engineering, Central Nippon Expressway Co., Ltd., Nagoya, Japan; 4 Institute of Industrial Science, the University of Tokyo, Tokyo, Japan; University Zurich, SWITZERLAND

## Abstract

**Objectives:**

In the brain, the mechanisms of attention to the left and the right are known to be different. It is possible that brain activity when driving also differs with different horizontal road alignments (left or right curves), but little is known about this. We found driver brain activity to be different when driving on left and right curves, in an experiment using a large-scale driving simulator and functional near-infrared spectroscopy (fNIRS).

**Research Design and Methods:**

The participants were fifteen healthy adults. We created a course simulating an expressway, comprising straight line driving and gentle left and right curves, and monitored the participants under driving conditions, in which they drove at a constant speed of 100 km/h, and under non-driving conditions, in which they simply watched the screen (visual task). Changes in hemoglobin concentrations were monitored at 48 channels including the prefrontal cortex, the premotor cortex, the primary motor cortex and the parietal cortex. From orthogonal vectors of changes in deoxyhemoglobin and changes in oxyhemoglobin, we calculated changes in cerebral oxygen exchange, reflecting neural activity, and statistically compared the resulting values from the right and left curve sections.

**Results:**

Under driving conditions, there were no sites where cerebral oxygen exchange increased significantly more during right curves than during left curves (*p* > 0.05), but cerebral oxygen exchange increased significantly more during left curves (*p* < 0.05) in the right premotor cortex, the right frontal eye field and the bilateral prefrontal cortex. Under non-driving conditions, increases were significantly greater during left curves (*p* < 0.05) only in the right frontal eye field.

**Conclusions:**

Left curve driving was thus found to require more brain activity at multiple sites, suggesting that left curve driving may require more visual attention than right curve driving. The right frontal eye field was activated under both driving and non-driving conditions.

## Introduction

Driving a vehicle is a complex activity in which more than 90% of information is obtained visually [1, 2]. It therefore requires not only motor control but also higher cognitive activity. In brain research on actual car driving, brain activity has been found to occur in multiple sites including the prefrontal cortex, motor related areas, and the parietal cortex [3]. In addition, enhanced brain activity in the prefrontal cortex and the motor related cortex have been reported in response to rapid changes in vehicle velocity [4]. However, differences in brain activity arising from road alignment have not been reported.

Differences in road alignment include differences between left and right curves. It has been reported that during cornering, a driver’s line of sight is likely to be directed to the inside of the curve [5, 6]. That is, the driver’s attention during a left curve is significantly directed to the left space, and attention during a right curve is significantly directed to the right space. The mechanisms of attention to the right and left are reported to differ hemispherically. Kinsbourne (1977) [7] found that the left and right cerebral hemispheres function in directing the attention to the opposite space, but left hemisphere attention to the right space is stronger than right hemisphere attention to the left space. Heilman et al. (1980) [8] and Kashiwagi et al. (1990) [9] suggest that the left hemisphere directs attention to the right space, but right hemisphere directs the attention to both spaces. The above studies suggest the hypothesis that left and right curves may give rise to differences in driver brain activity.

Driver brain activity has conventionally been studied using functional brain imaging and a driving simulator. With positron emission tomography (PET) or functional magnetic resonance imaging (fMRI), subjects are in a supine position, obviously unlike the posture of actual driving [10–15]. The simulator in these studies is also operated differently from operation of an actual vehicle; for example, by a push button [11] or a joystick [12, 13]. Studies using electroencephalography (EEG) and functional near-infrared spectroscopy (fNIRS) have been performed with subjects in a situation and posture resembling that of actual driving. In an EEG study, less activity in the right lateral prefrontal cortex was reported when driving faster than the speed limit [16]. In fNIRS research, measurement has been confined to the frontal lobe, and increases in oxyhemoglobin have been reported to be greater during driving than while stopped [17]. Increased oxyhemoglobin in the frontal lobe while driving under the direction of another person has also been shown to be greater than when driving from memory [18], but no brain studies have focused on road alignments and directly compared driving on left and right curves with the participants in the actual driving position. Whether differences in brain activity arising from road alignment are caused by differences in visual processing, driving operation, or other factors is also unknown. Experiments using driving simulation may be a valid way of separating out the factors of visual processing and driving operation from other factors that may cause brain activity while driving, because the road environment and visual information can be controlled.

Accordingly, we decided to test the hypothesis that driver brain activity would be different when driving on left and right curves using fNIRS and a large-scale 3-screen driving simulator to best replicate the actual driving environment. We report here our findings that the sites of brain activity differed when the participants drove on left and right curves.

## Materials and Methods

### Participants

Fifteen healthy adults (31.4 years old, standard deviation [SD] 4.4; 8 males and 7 females) participated in this study. Using the Edinburgh Handedness Inventory, we confirmed that 12 participants were right-handed and 3 were left-handed. Edinburgh Handedness Inventory scores were +98.3 (SD 4.0) for the right-handed group and -64.9 (SD 25.9) for the left-handed group. The 3 left-handed participants were all male. None of the participants had any history of neurological disease. They all had driving licenses, but their driving experience and accident histories were random. The experimental procedure is in accordance with the principles of the Declaration of Helsinki, and was reviewed and approved by the ethics committee at KatoBrain Co., Ltd. All participants received full explanation of the procedures and provided written informed consent for participation in the study.

### Experimental driving conditions

**Fig 1:**
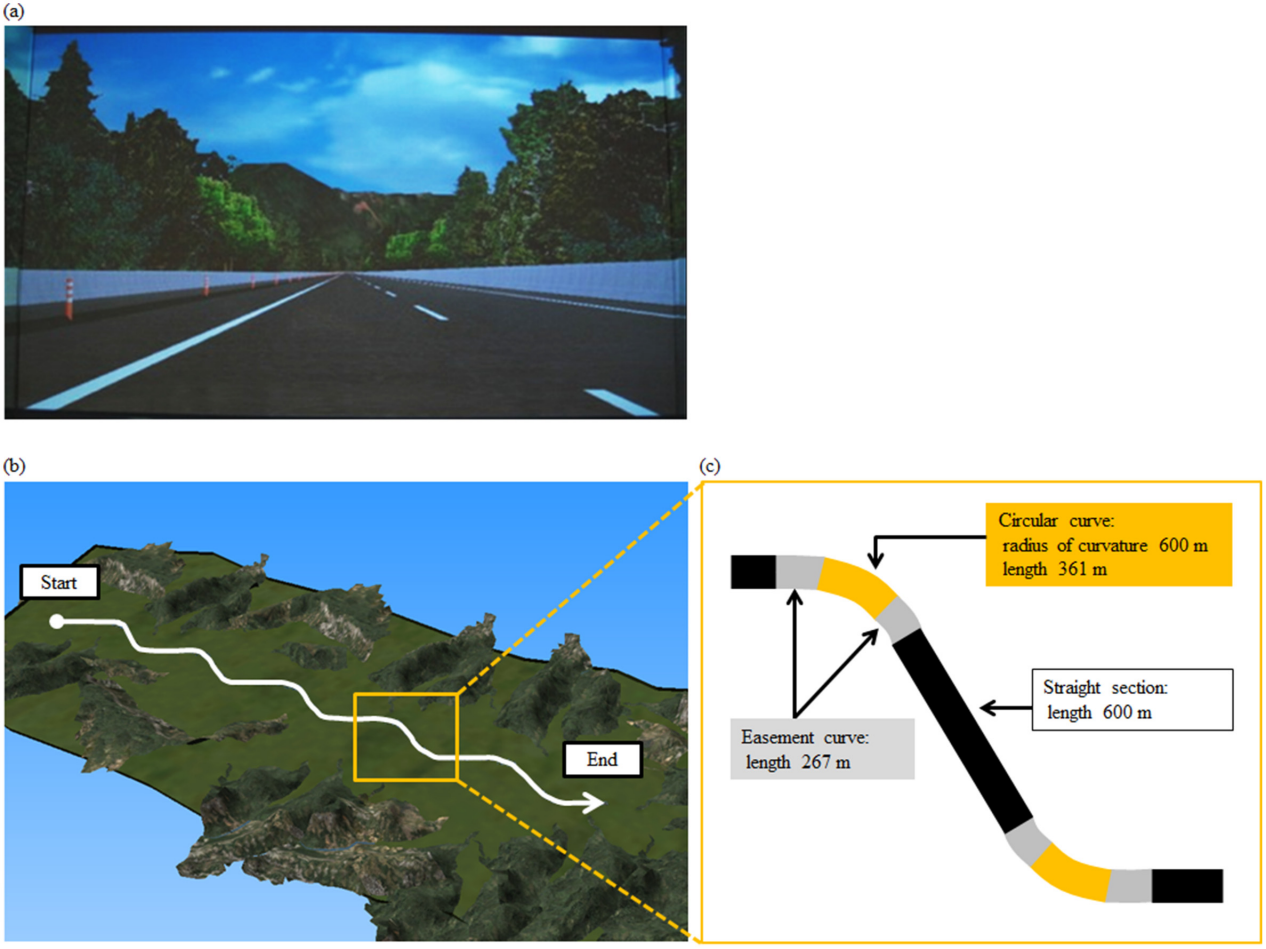
Experimental course. (a) Starting point. (b) Overview of entire course. (c) Schematic view of a left curve and a right curve. The course comprised 5 of these right/left curve sections.

An experimental expressway course was created for the simulator using the program software Multigen Creator (Presagis Canada Inc., Canada; Fig 1a and 1b). Driving on curves on an expressway involves a smaller range of driving operations than on smaller local roads, and there are fewer changes in speed and visual stimuli on an expressway. We therefore believed that differences in brain activity related to left and right curve driving could be more easily and directly detected in expressway driving.

The experimental course was two lanes wide. Its total length was 15,950 m and it included five sets of left and right curves (Fig 1b). 600 m straight sections were placed between the left and right curves to avoid possible brain activity related to driving the previous curve. Each curve length was 895 m and comprised one circular curve (length 361 m) with a radius of curvature of 600 m, and two easement curves (length 267 m) connecting the straight part of the course to the circular curve (Fig 1c). Barrier heights, lane widths and the like replicated those of the New-Tomei Expressway, and cross slopes were 0–2.5% in the straight sections and 2.5–8.0% in the curve sections. There were no longitudinal slopes.

### Procedures

The participants performed the experimental tasks in a sitting position, and the seat was positioned so that the brake pedal and the accelerator pedal could be easily operated. Before the experiment, they were allowed to practice drive the experimental course, so that they were comfortable with the simulator. The experimenter also explained, before the experiment, that the experiment could be interrupted if the participants experienced any driving simulator sickness (e.g. motion sickness or dizziness) during the task. None of the participants asked to stop. The participants drove in the left lane in accordance with Japanese law.

For the experiment, each participant drove the course once (driving conditions) and then traveled the course once without driving (non-driving conditions). In the driving part of the experiment, the participants were instructed to operate the accelerator pedal and the steering wheel to drive the course at a speed of 100 km/h. Average time for driving the right curve sections was 32.3 s (SD 1.0), and the left curve sections, 32.3 s (SD 0.9). Average speed in the right curve sections was 99.9 km/h (SD 2.9), and in the left curve sections, 99.7 km/h (SD 2.6). Differences in driving speed and time between the left and right curve sections thus were not significant (speed: *t* [145] = 0.520, *p* = 0.604; time: *t* [145] = -0.470, *p* = 0.639). The average steering angle in the right curve sections was 8.6° (SD 1.4), and in the left curve sections, 8.4° (SD 1.3). The maximum steering angle in the right curve sections was 15.3° (SD 2.4), and in the left curve sections, 14.9° (SD 2.0). The average steering angles and maximum steering angles thus did not differ significantly between the left and right curve sections (average steering angles: *t* [145] = 0.675, *p* = 0.501; maximum steering angles: *t* [145] = 1.096, *p* = 0.275), confirming that steering in the right and left curve sections was symmetrical.

In the non-driving part of the experiment, the participants simply watched while moving images from the log data recorded during the driving part of the experiment were projected on the screen. Environmental sounds such as engine and running sound and platform motion linked to the log were also reproduced in the same way as during the driving part of the experiment, so that the only difference between the driving and non-driving conditions was whether the participant operated the vehicle using the accelerator and the steering wheel.

After the experiments (both driving and non-driving), the participants were asked orally which curves (left or right) felt easier, and they reported after reflection no significant bias in difficulty of driving the left and the right curves: 7 participants (47%) felt the left curves were easier, 5 participants (33%) felt the right curves were easier, and 3 participants (20%) reported no difference (χ^2^ [2] = 1.6, *p* = 0.45). Under non-driving conditions, 14 participants (93%) felt no difference (χ^2^ [2] = 24.4, *p* = 0.000).

Each participant was also asked after the end of each task if he or she had experienced motion sickness or dizziness (on a scale increasing from 1 to 5). Their average scores (all participants) were 1.1 (SD 0.3) when driving and 1.2 (SD 0.4) when not driving, indicating that simulator sickness during the task was not significant.

#### Driving simulator

**Fig 2:**
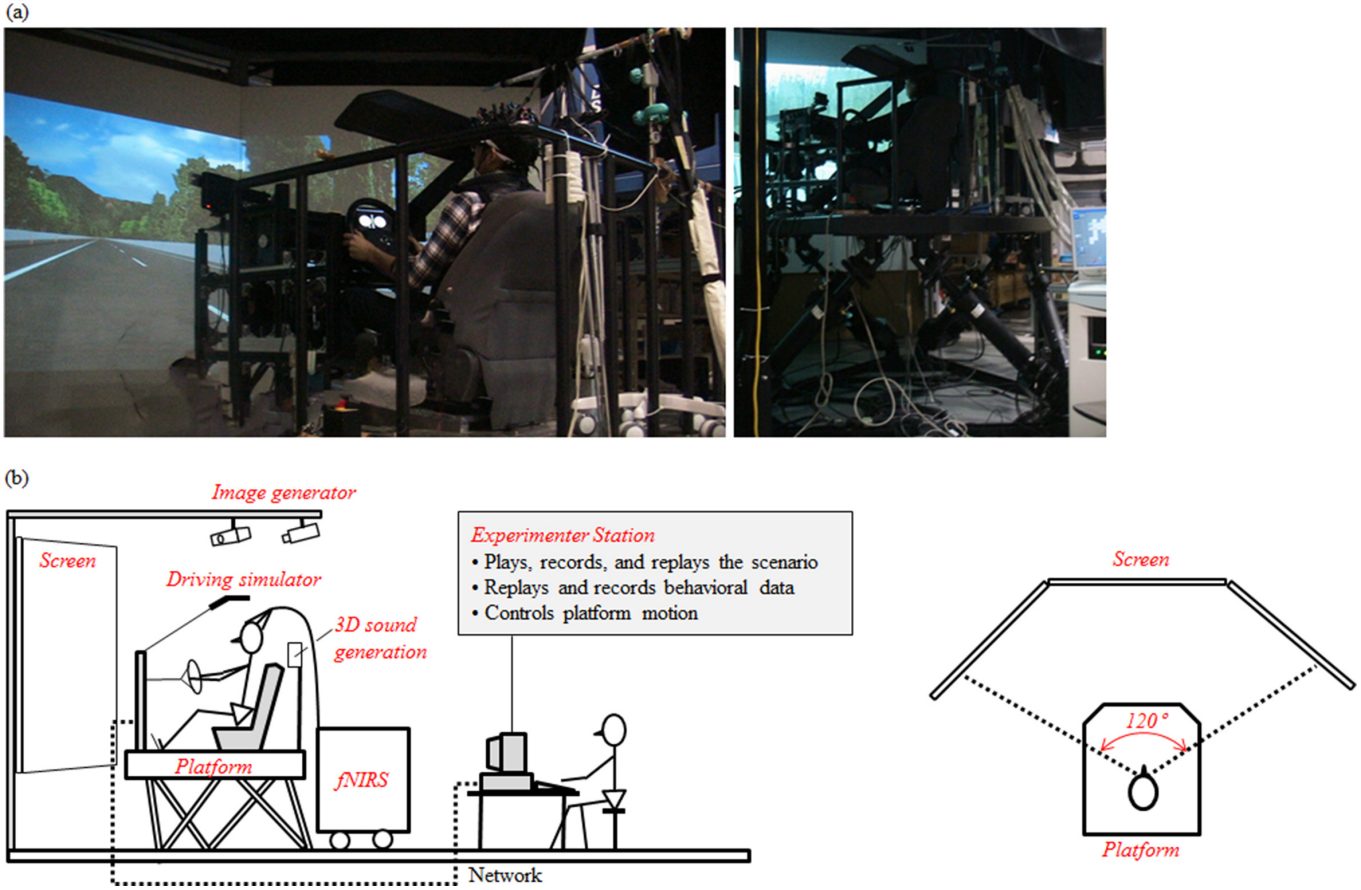
Driving simulator system. (a) Photos of the experimental landscape and setup. (b) Schematic views of the experimental setup.

The experiments were performed using a large scale driving simulator (Mitsubishi Precision Company Limited, Japan). This provided a seat, steering wheel, brake pedal and accelerator pedal used in an actual vehicle, and reproduced the sounds, visual fields, and dynamic changes of the platform accompanying vehicle operation in order to provide an environment as close as possible to that of actual vehicle operation. The driving scenario was projected by 3 projectors on a screen with a horizontal field of view of approximately 120 degrees. The driver’s field of view was limited by driver’s seat pillars so as not to extend beyond the image projected on the screen. The acoustic environment of driving (engine sound, wind noise and the like) was supplied from 2 speakers in the back of the driver’s seat. Platform motion due to steering was reproduced using a six-degree-of-freedom Stewart platform (Fig 2).

#### fNIRS data acquisition

**Fig 3:**
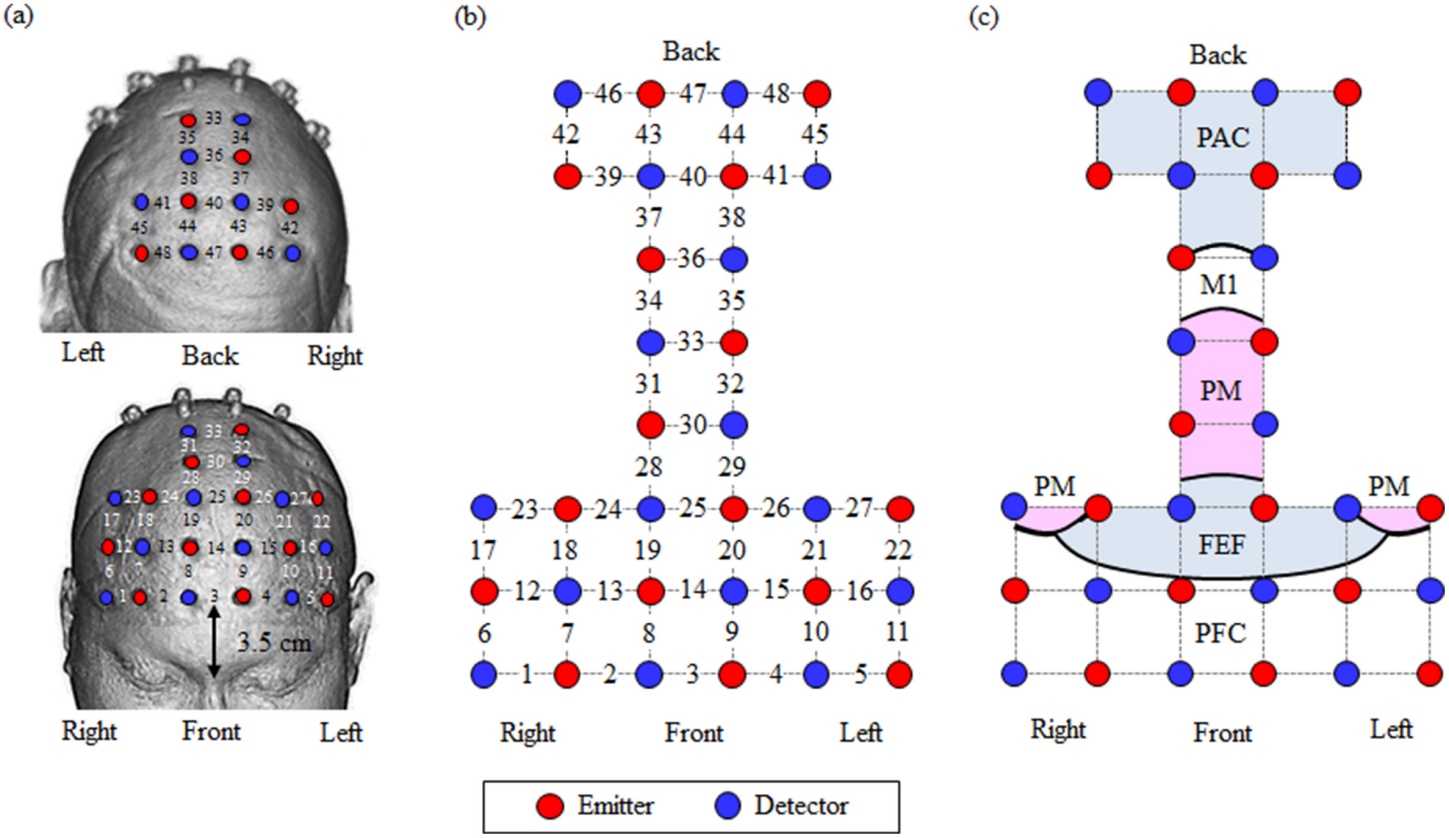
Relationship between channel configuration / probe placement and the underlying brain sites. (a) Probes mounted on the head. (b) Probe arrangement and measurement channel numbers. (c) Relationship between brain regions and measurement channels were confirmed using MRI. PFC: prefrontal cortex (including superior and middle frontal gyrus). PAC = parietal association cortex, M1 = primary motor cortex, PM = premotor cortex, FEF = frontal eye field, and PFC = prefrontal cortex.

Brain activity was monitored using multi-channel fNIRS (FOIRE-3000, Shimadzu Corporation, Japan). The fNIRS apparatus was placed in back of the driving simulator, as shown in Fig 2b. Probe holders (made by ourselves) were positioned with reference to the nasion and the inion. The measurement targets were the frontal cortex, the motor cortex and the parietal cortex. There were 48 measurement channels in all. Concentration changes in oxyhemoglobin and deoxyhemoglobin (ΔOxyHb and ΔDeoxyHb) were sampled at 70 ms. Event markers were input to the apparatus when the simulation passed through the starting point of each easement curve before a curve and again at the end point of the easement curve after the curve. After the experiment, registration markers were placed on the probe sites and 3D-T2 MRI images (3 Tesla) were obtained of all participants to confirm the measurement channel positions (Fig 3).

### Analysis

Each curve was counted as a trial for a total of 75 trials (15 participants x 5 trials) for left and right, driving and non-driving, but because of skipped markers, only 74 left curve trials and 73 right curve trials were analyzed for both conditions.

**Fig 4:**
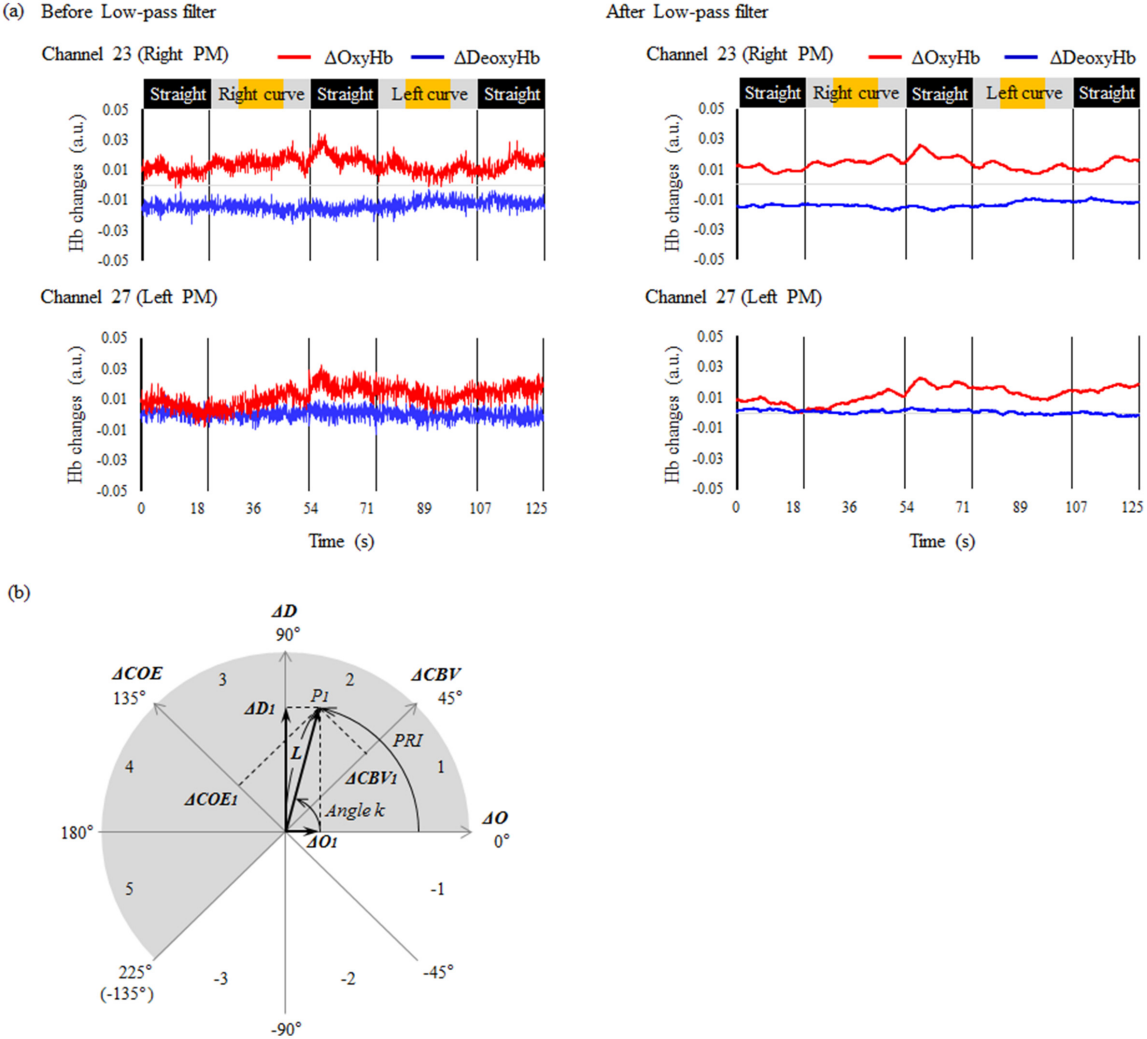
Time-series waveforms of ΔOxyHb and ΔDeoxyHb, and a *ΔO*/*ΔD* vector plane. (a) Raw waveform data for changes in oxyhemoglobin and deoxyhemoglobin (ΔOxyHb and ΔDeoxyHb) from the right PM (channel 23) and the left PM (channel 27). These channels are symmetrical regions of the cerebral cortex. Frequencies of more than 0.1 Hz were removed from the raw data using a low-pass filter. Straight sections of the course are indicated in black, easement curves in gray, and circular curves in orange. PM = premotor cortex. (b) Vector trajectories on this polar coordinate plane make it possible to visually evaluate relationships between changes in the cerebral blood volume vector component (***ΔCBV***) and the cerebral oxygen exchange vector component (***ΔCOE***).

Raw ΔOxyHb and ΔDeoxyHb data obtained using fNIRS were processed using a low-pass Butterworth filter (Fig 4a). Components with higher than 0.1 Hz frequencies were removed from the raw data, based on Mayer wave frequencies [19] and previous research showing that the main causes of fluctuation of obtained optical signals are oscillations of 0.1 Hz resulting from regional cerebral blood flow [20]. The starting point of the easement curve was taken as zero in calculating ΔOxyHb and ΔDeoxyHb to measure event-related reactions. Analysis was performed by the vector analysis method, using an orthogonal vector plane with a ***ΔO*** axis and a ***ΔD*** axis, representing the vector components of change in oxyhemoglobin and change in deoxyhemoglobin, respectively [21, 22]. As shown in Fig 4b, this polar coordinate plane has as vector components the 4 indicators ***ΔO***, ***ΔD***, ***ΔCOE*** (change in cerebral oxygen exchange vector component) and ***ΔCBV*** (change in the cerebral blood volume vector component) [23, 24]. ***ΔO*** and ***ΔD*** are obtained from the measured values of ΔOxyHb and ΔDeoxyHb, and ***ΔCOE*** and ***ΔCBV*** are calculated from ***ΔO*** and ***ΔD***, using Eqs (1) and (2):

$$\Delta COE=\frac{1}{\sqrt{2}}\left(\Delta D-\Delta O\right)$$ (1)

$$\Delta CBV=\frac{1}{\sqrt{2}}\left(\Delta D+\Delta O\right)$$ (2)

As Eqs (1) and (2) show, the vector component ***ΔCBV*** is orthogonal to ***ΔCOE***. Eqs (3) and (4) show that ***ΔCBV*** is different from concentration changes in total-hemoglobin (ΔtotalHb), which is calculated as the sum of ΔOxyHb and ΔDeoxyHb.

ΔtotalHb=ΔOxyHb+ΔDeoxyHb (3)

|ΔtotalHb|=|ΔOxyHb+ΔDeoxyHb|=|ΔO+ΔD| (4)

The relationship between ΔtotalHb and ***ΔCBV*** is represented by Eq (5).

$$\left|\Delta \text{totalHb}\right|=\sqrt{2}∙\left(\left|\Delta CBV\right|\right)$$ (5)

***ΔCOE*** is an indicator of changes in oxygenation in the blood vessels and reflects neural activity [21]. Increasing ***ΔCOE*** (from ***ΔCOE*** = 0) means that deoxygenation is occurring in the capillaries as a result of oxygen consumption by the nerve cells, and indicates hypoxia in the blood vessels. Decreasing ***ΔCOE*** means that oxygen-containing red blood cells are being supplied from the arteries, and indicates a high level of oxygenation in the blood vessels. ***ΔCOE*** shows a higher degree of precision as a physiological indicator of increased brain function than the conventionally used ***ΔO*** [4]. The combination of increasing ***ΔO*** and decreasing ***ΔD***, which was conventionally considered to indicate changing skin blood flow [25, 26], is not included in the phases showing increasing ***ΔCOE***. In addition, as shown in Fig 4b, the index ***ΔCOE*** is perpendicular to the index ***ΔCBV***. In other words, ***ΔCOE*** is not easily affected by changes in ***ΔCBV***.

We calculated average values of ***ΔCOE*** during left curve driving and right curve driving for both driving and non-driving trials, and created mapping images of these average ***ΔCOE*** values for left and right curve driving.

Average values of ***ΔCBV*** and ***ΔCOE*** were calculated every 3 seconds for 30 seconds from the starting point of the first easement part of each curve. Using these averages, vector trajectories for all 10 curve sections of the course were plotted on the vector plane. The phase of the vector for each section was determined from Eq (6), using the included angle *k* between the positive ***ΔO*** axis and the vector obtained by measurement:

$$∠k=\;Arc\;\text{tan}\left(\frac{\Delta D}{\Delta O}\right)=\;Arc\;\text{tan}\left(\frac{\Delta COE}{\Delta CBV}\right)+45{}^{\circ}\;\left(-135{}^{\circ}≦∠\;k≦225{}^{\circ}\right)$$ (6)

*k* represents the ratio of ***ΔCOE*** to ***ΔCBV***, and defines the degree of oxygen exchange [22]. *k* = 0 is on the positive ***ΔO*** axis, and *k* increases in the counterclockwise direction. *k* > 0 reflects hypoxia or deoxygenation and indicates increased neural activity. *k* decreases in the clockwise direction, and *k* < 0 reflects hyperoxidation and indicates decreased neural activity. From the ratios between ***ΔO***, ***ΔD***, ***ΔCBV*** and ***ΔCOE***, the state of neural activity can be divided into eight phase types on the vector plane (Fig 4b and Table 1) [23]. Increases in *k* (0–225°) fall into Phases 1 through 5, while decreases in *k* (-135–0°) fall into Phases -1 through -3. Phases 1 through 5, which show increased ***ΔD*** or increased ***ΔCOE***, are considered to show increased brain activity. A relationship between phase distribution and oxygen saturation has also been reported [27]. In Phases 3 and 4 (indicating hypo-oxygenation), oxygen saturation decreases independently of the measurement baseline, and in Phases -1 and -2 (indicating hyper-oxygenation), oxygen saturation increases independently of the measurement baseline. In contrast, Phases -1 through -3 (decreasing ***ΔCOE*** and ***ΔD***), can be considered to show almost no increase in brain activity.

**Table 1:** Phase classification of hemodynamic responses.

Phase	Description	Relationships between the 4 indicators
Phase 1	Hyperoxia/Hyperemia	0 < *ΔD* < *ΔO*, *ΔCOE* < 0 < *ΔCBV*
Phase 2	Hypoxia/Hyperemia	0 < *ΔO* < *ΔD*, 0 < *ΔCOE* < *ΔCBV*
Phase 3	Hypoxia/Hyperemia	*ΔO* < 0 < *ΔD*, 0 < *ΔCBV* < *ΔCOE*
Phase 4	Hypoxia/Ischemia	*ΔO* < 0 < *ΔD*, *ΔCBV* < 0 < *ΔCOE*
Phase 5	Hypoxia/Ischemia	*ΔO* < *ΔD* < 0, *ΔCBV* < 0 < *ΔCOE*
Phase -1	Hyperoxia/Hyperemia	*ΔD < 0 < ΔO*, *ΔCOE* < 0 < *ΔCBV*
Phase -2	Hyperoxia/Ischemia	*ΔD* < 0 < *ΔO*, *ΔCOE* < *ΔCBV* < 0
Phase -3	Hyperoxia/Ischemia	*ΔD* < *ΔO* < 0, *ΔCBV* < *ΔCOE* < 0

A scalar *L* from the vector origin to given coordinates on the vector plane shows the amplitude of the vector and represents the amount of hemoglobin variation. *L* can be calculated from Eq (7):

$$L\;=\;\sqrt{{\left(\Delta O_{1}\right)}^{2}+{\left(\Delta D_{1}\right)}^{2}}\;=\;\frac{1}{\sqrt{2}}\sqrt{{\left(\Delta D_{1}-\Delta O_{1}\right)}^{2}+{\left(\Delta D_{1}+\Delta O_{1}\right)}^{2}}\;=\;\sqrt{{\left(\Delta COE_{1}\right)}^{2}+{\left(\Delta CBV_{1}\right)}^{2}}$$ (7)

Phase-associated response intensity (*PRI*; Fig 4b) can be visualized as the arc, in a positive or negative direction from ***ΔO*** = 0, created by the angle *k* and the scalar *L*. *k* and *L* are separate brain activity indices that move in conjunction with each other. Eq (8) shows the calculation of *PRI*. *k* ^*rad*^ is in radians:

$$Phase\text{-associated}\;\text{response}\;\text{intensity}\;\left(PRI\right)=\;k^{rad}・L$$ (8)

*PRI* reflects both the angle *k* and the scalar *L*, which are calculated from the 4 indices (***ΔO***, ***ΔD***, ***ΔCBV*** and ***ΔCOE***) measured by fNIRS. Increases in *PRI* show increasing intensity of the hemodynamic responses involved in oxygen exchange, and decreases in *PRI* show decreasing intensity.

### Statistics

An independent t-test was used to compare average left and right curve ***ΔCOE*** values under driving and non-driving conditions. In this analysis, the trial was adopted as smallest statistical unit, as the brain responses involved in different trials by the same subject were not necessarily considered to be equivalent. To examine the effect of handedness and gender on the average ***ΔCOE*** values in the left and right curves, we used two-way analysis of variance (two-way ANOVA), with handedness and gender as fixed factors. As all 3 left-handed participants were male, interactions between handedness and gender could not be examined; we therefore examined only the main effects.

At sites where comparison of average left and right curve ***ΔCOE*** values showed significant differences, vector differences by segment under driving and non-driving conditions were compared using MANOVA, with ***ΔCOE*** and ***ΔCBV***, which are vector components, as the dependent variables. Values used in this analysis were average values of ***ΔCBV*** and ***ΔCOE***, calculated every three seconds for 30 seconds from the starting point of the first easement part of each curve. Significant difference between vectors in this analysis refers to scalar comparisons in the case of comparing vectors within the same phase, but when vectors in different phases are compared, not only vector strength, but also difference in phase is reflected [23].

In the segment of maximum average *L* values (measured every three seconds from the starting point of each curve), left and right curve *PRI* values were calculated and compared using the independent t-test.

Statistical analysis was performed using the software SPSS (SPSS Statistics Version 22.0, IBM, Japan). All multiple testing was performed as exploratory analysis. In all the tests, *p* < 0.05 was considered significant.

## Results

### Driving

In the driving trials, no sites showed significantly more brain activity during right curves than during left curves. Significantly greater activity was detected in the left curves than in the right curves in 4 areas of the brain.

#### Left curves (driving)

**Fig 5:**
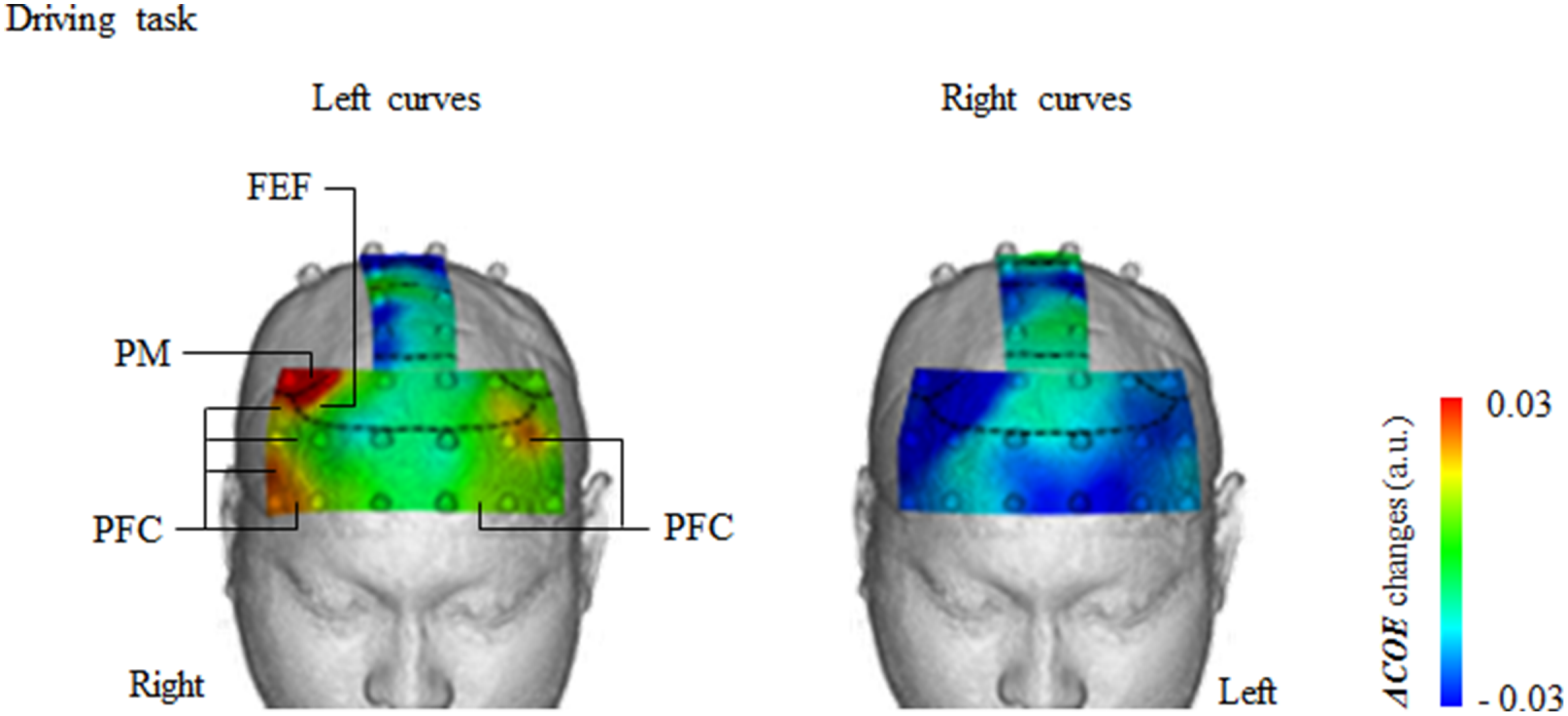
Brain activity (*ΔCOE*) during left and right curve driving. Under driving conditions, *ΔCOE* increased more in left curves than in right curves in four areas of the brain: right PM (premotor area), right FEF (frontal eye field) and bilateral PFC (prefrontal cortex). a.u. = arbitrary unit.

As Fig 5 shows, ***ΔCOE*** showed significantly greater increases in 4 areas of the frontal lobe during left curve driving than during right curve driving: the right premotor cortex (PM, channel 23), the right frontal eye field (FEF, channel 18) and the bilateral prefrontal cortex (right PFC, channels 1, 6, 12 and 17; left PFC, channels 4 and 16) (right PM: *t* [145] = 2.87, *p* = 0.005, *d* = 0.47; right FEF: *t* [145] = 2.57, *p* = 0.011, *d* = 0.42; right PFC: *t* [145] = 2.42–2.86, *p* = 0.005–0.017, *d* = 0.40–0.47; left PFC: *t* [145] = 2.05–2.66, *p* = 0.009–0.042, *d* = 0.34–0.44).

**Fig 6:**
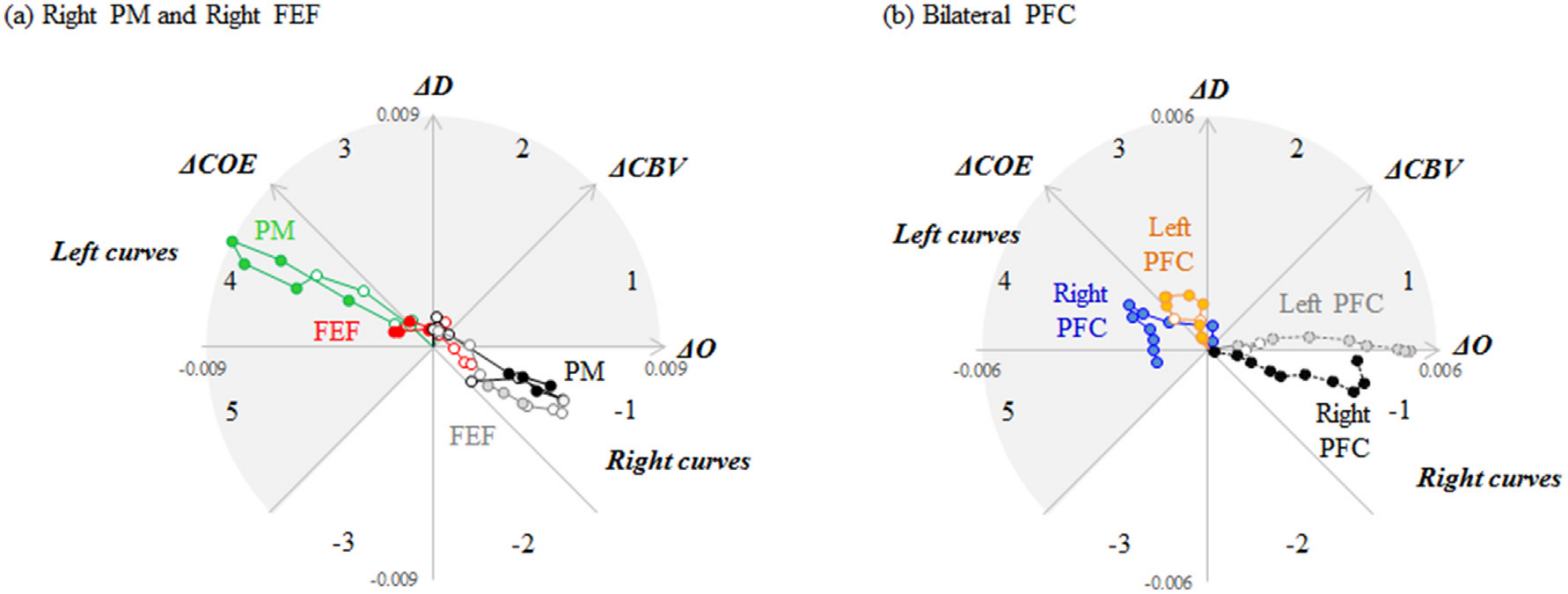
Vector tracks during left and right curve driving. Vector tracks from the 4 sites of activity plotted every 3 seconds during left and right curve driving, beginning 30 seconds from the start of the easement curve. (a) Vector tracks from the right PM (premotor cortex, channel 23) and the right FEF (frontal eye field, channel 18). In both areas, the left curve vector tracks are mainly in Phase 4, and the right curve vector tracks are in Phase -1. Significant differences between left and right curves are indicated by solid color dots. Significant differences were detected from 9.1 to 27.0 s in the right PM, and from 9.1 to 18.0 s in the right FEF (right PM: *F* [2, 144] = 4.210–7.266, *p* = 0.001–0.017, $\eta_{p}^{2}$ = 0.06–0.1; right FEF: *F* [2, 144] = 3.636–4.265, *p* = 0.016–0.029, $\eta_{p}^{2}$ = 0.05–0.06). (b) Vector tracts from the right PFC (prefrontal cortex, channels 1, 6, 12 and 17 [average]) and the left PFC (channels 4 and 16 [average]). Left curve vector tracks from the right PFC are in Phases 3 and 4, and right curve vector tracks are in Phase -1. Left curve vector tracks from the left PFC are in Phase 3, and right curve vector tracks are in Phase 1. Significant differences between left and right curves are indicated by solid color dots. Significant differences were detected from 0.0 to 30.0 s in the right PFC, and from 0.0 to 3.0 s and 9.1 to 30.0 s in the left PFC (right PFC: *F* [2, 585] = 3.786–18.77, *p* = 0.000–0.023, $\eta_{p}^{2}$ = 0.01–0.06; left PFC: *F* [2, 291] = 3.318–6.585, *p* = 0.002–0.038, $\eta_{p}^{2}$ = 0.022–0.043).

Fig 6 shows vector changes during left and right curves in the 4 regions where there was significantly greater activation in the left curves. In all 4 regions, the left curve vectors advanced into the phases opposite those of the right curve vectors. The angle *k* of the left curve vectors increased and peaked in Phases 3 and 4. In other words, the amount of change in the ***ΔCOE***-axis direction was greater than that in the ***ΔCBV***-axis direction at these sites. This indicates a strong pattern of oxygen metabolism.

In contrast, the right curve vectors advanced into Phases 1 and -1 (decreasing ***ΔCOE***) in all 4 sites, indicating an absence of strong oxygen consumption.

Among the 4 regions that were activated significantly in the left curve sections, differences in brain activity by dominant hand were detected only in the right PFC (channel 1) (*F* [1, 71] = 11.220, *p* = 0.001, $\eta_{p}^{2}$ = 0.14). Differences in brain activity according to dominant hand were also observed in the bilateral prefrontal cortex (right: channel 2; left: channel 5) and in the M1 (channel 36). ***ΔCOE*** in the bilateral prefrontal cortex increased significantly more in the right-handed group than in the left-handed group (right: *F* [1, 71] = 12.876, *p* = 0.001, $\eta_{p}^{2}$ = 0.15; left: *F* [1, 71] = 6.813, *p* = 0.011, $\eta_{p}^{2}$ = 0.09). ***ΔCOE*** in the M1 increased significantly more in the left-handed group than in the right-handed group (*F* [1, 71] = 6.758, *p* = 0.011, $\eta_{p}^{2}$ = 0.09)

Among the 4 sites showing significant activity in left curves, differences in brain activity by gender were detected only in the right PFC (channel 1). ***ΔCOE*** in the right PFC increased in the both groups, but significantly more in the male group than in the female group (*F* [1, 71] = 5.894, *p* = 0.018, $\eta_{p}^{2}$ = 0.08). Differences in brain activity according to gender were observed in the right parietal associate cortex (PAC, channel 46) (right PAC: *F* [1, 71] = 9.072, *p* = 0.004, $\eta_{p}^{2}$ = 0.11). ***ΔCOE*** in the right PAC increased significantly more in the female group than in the male group.

#### Right curves (driving)

**Fig 7:**
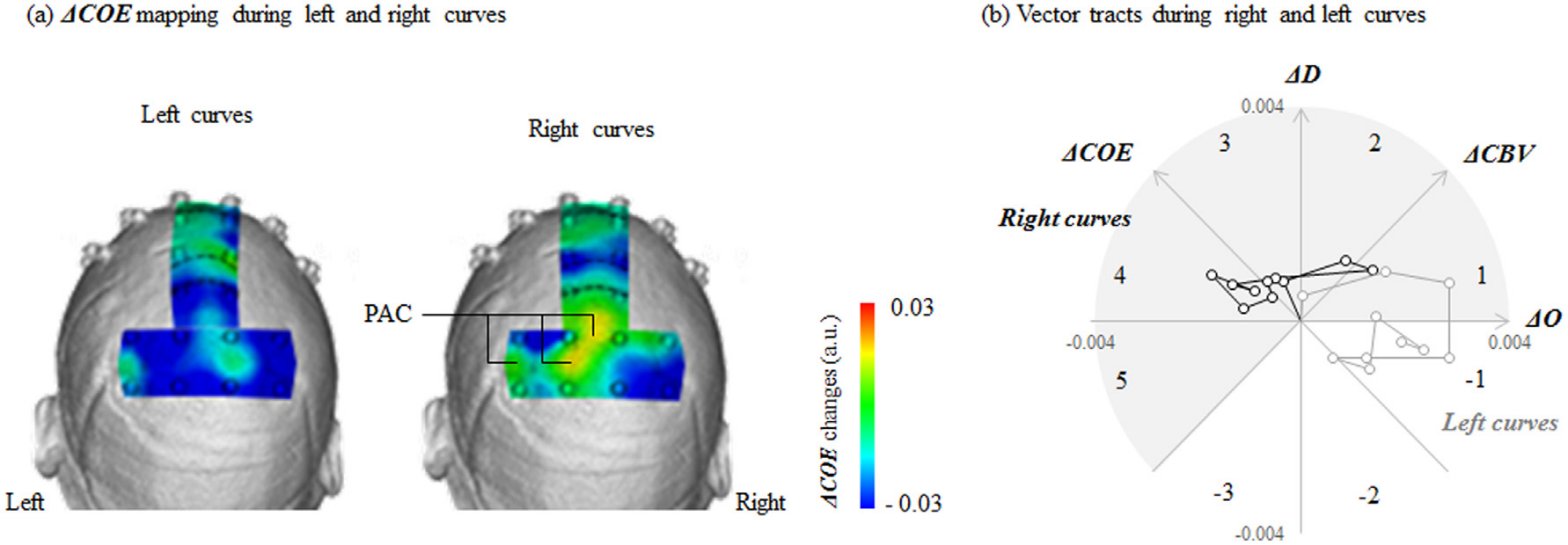
Brain activity (*ΔCOE*) in the PAC (parietal association cortex) during left and right curve driving. (a) Under driving conditions, *ΔCOE* showed the greatest increase in right curves in the left PAC. (b) Vector tracks during right and left curves in the left PAC (channels 40, 44 and 45 [average]) plotted every 3 seconds, beginning 30 seconds from the start of the easement curve. At *L* peak times, the right curve vector is in Phase 4, and the left curve vector is in Phase 1. a.u. = arbitrary unit.

At no site did ***ΔCOE*** increase significantly more during right curves than during left curves. ***ΔCOE*** increased during right curves only in the left parietal association cortex (PAC, channels 40, 44 and 45; Fig 7).

As for effects of handedness, no significant differences in ***ΔCOE*** according to dominant hand were observed in the left PAC. There was no region in which ***ΔCOE*** increased significantly more in the right-handed group than in the left-handed group. There was no region in which ***ΔCOE*** increased significantly more in the left-handed group than in the right-handed group.

As for possible effects of gender, ***ΔCOE*** in the left PAC (channel 38) increased significantly more in the female group than in the male group (*F* [1, 70] = 11.180, *p* = 0.001, $\eta_{p}^{2}$ = 0.14). The sites where differences in ***ΔCOE*** were detected according to gender, however, were not the same sites where differences in ***ΔCOE*** were detected according to left and right curves. Differences in gender thus did not affect the results of this study.

### Non-driving

**Fig 8:**
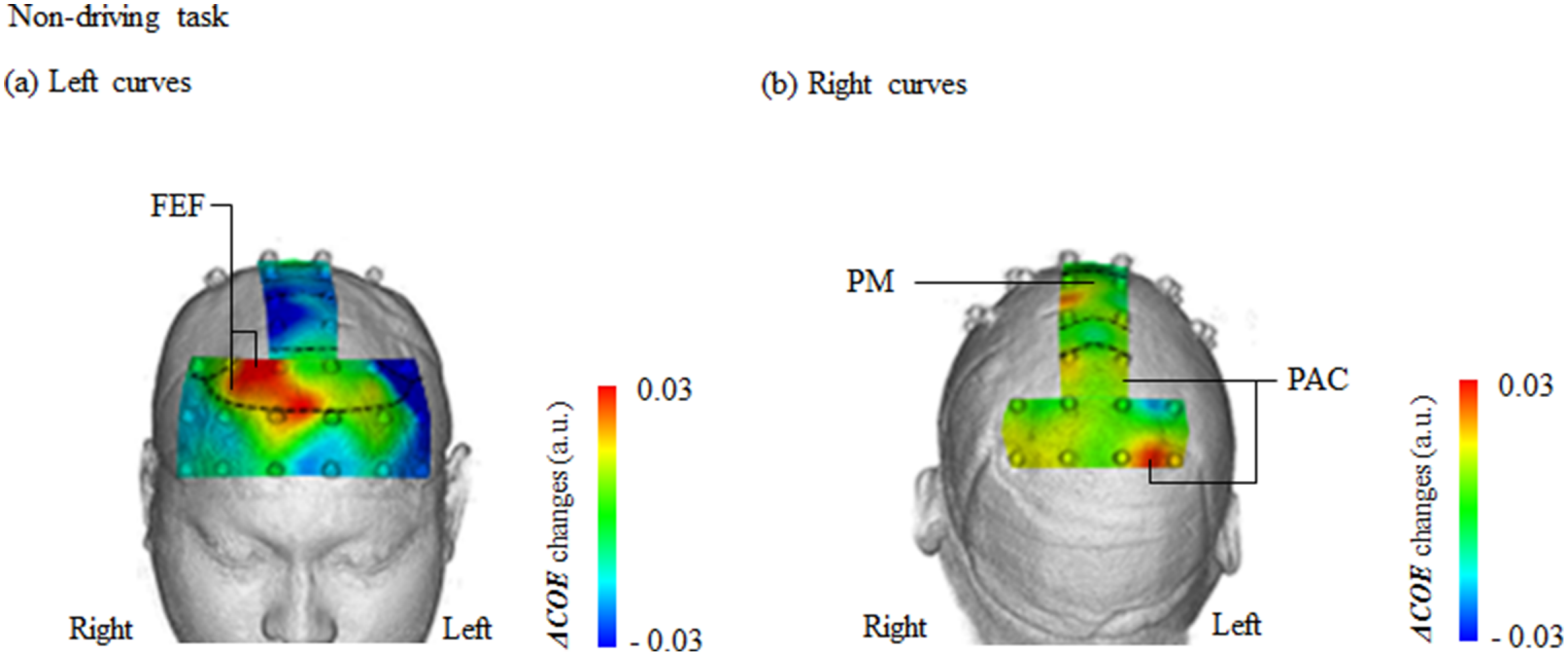
Brain activity (*ΔCOE*) during left and right curves under non-driving conditions. (a) Brain activity during left curves: activity in the right FEF (frontal eye field, channels 18 and 24) increased significantly more in the left curves. (b) Brain activity during right curves: activity in the medial PM (premotor area, channel 30) and right PAC (parietal association cortex, channels 37 and 46) increased significantly more in the right curves. a.u. = arbitrary unit.

Among the 4 regions where brain activity increased significantly in the left curves during the driving part of the experiment, only the right FEF showed significant activation under non-driving conditions [channels 18 and 24: *t* (145) = 2.20–2.26: *p* = 0.025–0.029, *d* = 0.36–0.37] (Fig 8). Significant activation in the three other regions (right PM and bilateral PFC) disappeared. In particular, activity in the right PM was significantly less from the start to the middle of the curve (0.0–6.0 s, 12.1–21.0 s; *F* [2, 145] = 3.109–3.850, *p* = 0.023–0.476, $\eta_{p}^{2}$ = 0.04–0.05). No sites showed significant activation in the left curves under non-driving conditions alone.

In the right curves, the sites where ***ΔCOE*** increased significantly more than in the left curves were the right PAC (channels 37 and 46) and the medial PM (channel 30) (right PAC: *t* [145] = 1.99–2.67: *p* = 0.008–0.022, *d* = 0.38–0.44; medial PM: *t* [145] = 1.99: *p* = 0.049, *d* = 0.33) (Fig 8). ***ΔCOE*** decreased in these sites under driving conditions.

### Comparison of brain activity during curve driving

Marked increases in ***ΔCOE*** were detected in curve driving in a total of 5 brain sites, and in 4 of these (right PM, right FEF, and bilateral PFC), significant increases were detected in the left curves. In the right curves, the only site where an increase was detected was the left PAC, but the amount of increase detected was not significant.

Comparison of amounts of brain activity in these five regions using vector analysis gives the vector peak time zones shown in Table 2. At all sites showing significant left curve activation, *L* peaked in a time zone corresponding to the middle part of the curve (12.1–18.0 s). Peak activation during right curves in the PAC occurred later than it did during left curves (18.1–21.0 s).

**Table 2:** Characteristic vectors at vector peak time zones under driving conditions.

tCurve	Brain region	Peak times for *L*	*L*	*k* (degrees)
Left	Right PM	15.1–18.0 s	0.0088	152.5
	Right FEF	12.1–15.0 s	0.0017	159.0
	Right PFC	12.1–15.0 s	0.0023	151.0
	Left PFC	15.1–18.0 s	0.0018	129.6
Right	Left PAC	18.1–21.0 s	0.0019	153.1

In the peak time zones, values of *k* ranged from 129.6 to 159.0 degrees in these 5 brain regions; namely, within a range of 30.4 degrees. The fact that the vectors changed in the direction of the ***ΔCOE*** axis shows that ***ΔCOE*** variation was greater than ***ΔCBV*** variation, indicating a pattern of strong oxygen metabolism.

**Fig 9:**
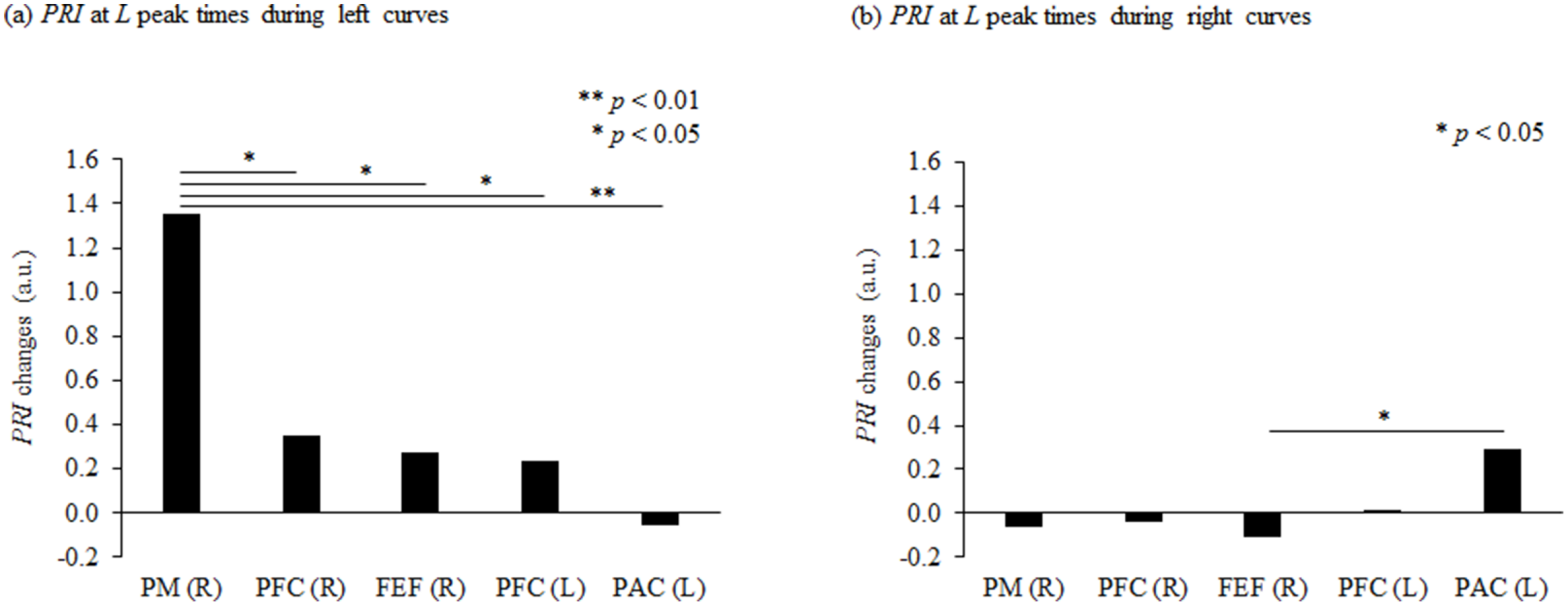
Comparison of *PRI* during curve driving in different sites of the brain. *PRI* (phase-associated response intensity) in the *L* peak time zones were compared between sites: (a) during left curve driving and (b) during right curve driving. Asterisks (*) indicate significant differences between the sites. PM = premotor area, PFC = prefrontal cortex, FEF = frontal eye field, and PAC = parietal association cortex. During left curve driving, activity was greatest in the right PM, and it was greater in the right frontal lobe than in the left hemisphere. During right curve driving, activity was greatest in the left PAC, followed by the left PFC, and activity was greater in the left frontal lobe than in the right hemisphere. a.u. = arbitrary unit.

*k* and *L* differed among the five regions, and the sites of maximum *k* and maximum *L* were not the same. We therefore compared *PRI* values to rank the sites in order of strength of brain activity (Fig 9). *PRI* was significantly higher in the right PM than in the 4 other regions in left curve driving; in other words, the greatest amount of brain activity in left curve driving was in the right PM (*F* [4, 365] = 6.361, *p* = 0.000–0.032, *d* = 0.37–0.57). This was followed in decreasing order by the right PFC, the right FEF and the left PFC. *PRI* decreased in the left PAC in left-hand curves, indicating that oxygen metabolism did not increase. The right frontal lobe thus showed the most activity in left curve driving.

In right curve driving, *PRI* increased in the left PFC and in the left PAC. *PRI* in right curve driving was highest in the left PAC (*F* [4, 360] = 3.147, *p* = 0.043, *d* = 0.68, followed by the left PFC. *PRI* decreased in 3 areas of the right hemisphere: the right PM, the right PFC and the right FEF, showing that activity in right curve driving was greater in the left hemisphere.

## Discussion

This study showed that left curve driving required considerably more brain activity than right curve driving, despite the absence of significant differences in steering angles, corroborating the hypothesis that brain activity is different in left and right curve driving. We also observed differences in brain activity in left and right curves whether or not the participant was operating the vehicle.

### Right premotor cortex activity during left curve driving

Right PM activity increased more in left curves than in right curves under conditions of vehicle operation. *PRI*, which is a useful index of brain activity derived from all 4 indices ***ΔO***, ***ΔD***, ***ΔCBV*** and ***ΔCOE***, showed that activity in the right PM was greatest in left curve driving. This activity, however, disappeared under non-driving conditions, when significantly less right PM activity was observed in the mid-curve time zone than during driving, indicating that right PM activity was induced by vehicle operation. Significant activity in the right PM has been reported when operating an actual vehicle [3], supporting the results of the present experiment. PM activity increased particularly in the mid-curve time zones, and the vectors during those time zones were distributed in Phase 4. Strong neural activity could thus be considered to have occurred in the right PM.

Driving is a complex activity in which more than 90% of information is obtained by visual means [1, 2]. As we drive, we watch the road alignment to prepare for the next driving operation, and the PM is involved in preparation for movement [28–30]. The right PM is a site that is particularly involved in motor output due to visual information [31, 32]. The performance of adjustments based on visual information and preparation for movement can be considered to be an important factor in the greater PM activity observed during driving trials but not during non-driving trials. It has also been reported that the PM is involved in coordination of movement of the two hands [33]. PM activity may arise with the use of both hands to turn the steering as the driver follows the curves in the road.

### Bilateral PFC activation during left curve driving

PFC activity on both sides increased more in left curves than in right curves under driving conditions. Differences between left curve and right curve activity in the bilateral PFC disappeared under non-driving conditions, when driving operations were not performed, indicating that the activity was induced by driving operations. The greater activity observed in the bilateral PFC during left curves was observed throughout the curves; however, the amount of hemoglobin change was greatest in the mid-curve time zone. In the middle section of the curve, *k* was greater in the right PFC than in the left PFC, indicating that neural activity was stronger in the right PFC than in the left PFC. The significant activity observed in the PFC also occurred in a wide range over the right hemisphere.

Damage to the right frontal lobe causes left unilateral neglect [34, 35] and thus the right PFC can be considered to be involved in visual attention to the left space. Because the driver’s line of sight when entering and traversing a curve is likely to be toward the inside of the curve in the traveling direction [5,6], the drivers’ attention in the present experiment can be thought to be directed to the left visual field in a left curve, resulting in an increase in right frontal lobe activity. Furthermore, because the participants were traveling in the left lane in this study, forward visibility during a left curve is more limited than during a right curve, and left curves may therefore require more visual attention than right curves.

Differences in brain activity appeared according to gender and handedness in 1 of the 4 channels of the right PFC showing significant activity in left curve driving. There was significantly higher activity in the right-handed group than in the left-handed group, and significantly higher activity in the male group than in the female group. This suggests that handedness and gender affect the activity of the right PFC, which has been shown to be involved in decision making [36, 37] and spatial working memory [38, 39]. We were unable to analyze the interaction of gender and handedness, as the left-handed participants were all male. Future study is required to take into consideration not only the separate effects of gender and handedness but also their interaction.

### Right frontal eye field activation during driving and non-driving conditions

Significantly greater activity was observed in the right FEF during left curves than during right curves not only under driving conditions, but also under non-driving conditions, when the participant was able to see the direction of travel without operating the vehicle. In the right FEF during driving, the amount of hemoglobin conversion was greatest in the time zones around the middle of the curves, and the vectors during those zones time were distributed in Phase 4. Under non-driving conditions, the right FEF vectors from the mid-curve time zones were also distributed in Phase 4. Strong neural activity is likely to have occurred in the mid-curve time zones whether driving or not driving.

Significant FEF activity has been confirmed when driving an actual vehicle on an expressway [3,4], supporting the results of the present experiment. The possibility that right FEF activity occurs in passengers of a moving vehicle as well as in the driver is a new finding.

The FEF is known to be involved in eye movement [40, 41], and is responsible for vergence and saccades [42]. The right FEF in particular has been reported to be involved in saccades toward the left side [43].

It follows that, in this study, the activity in right FEF during the left curves can be thought to be induced by saccades toward the left side. The line of sight while traversing a curve is likely to be toward the inside of the curve in the traveling direction, whether or not one is operating a vehicle [6]. The change in line of sight towards the traveling direction is greater in the circular curve sections than in the easement curve sections, and thus the increase in right FEF activity in the mid-curve time zones is likely to be induced by the saccadic function.

### PAC activation during right curve driving

The PAC was activated during right curve driving. The left PAC and the median PAC were activated during the driving part of the experiment, and the right PAC was activated under non-driving conditions; that is, opposite hemispheres were activated under driving and non-driving conditions.

These results are supported by prior reports. Activity in the left PAC and median PAC under driving conditions have been reported to occur both in a driving simulator [15] and in driving an actual vehicle [3]. Right PAC activity under non-driving conditions has been reported to occur in visual event detection without vehicle operation [11].

The PAC is involved in processing visual attention accompanying eye movement [44]. In particular, the left PAC is thought to be activated by oscillatory saccades and involved in temporal shifts in attention [45]. Namely, activity of the left PAC during driving is likely to be involved in moment-by-moment attention shifts in response to changes in road alignment. A possible role of the right PAC in top-down processing and/or the preparation of upcoming eye movement has also been pointed out [45]. The video projected on the screen for the non-driving part of the experiment was recorded as the participant drove in the driving part of the experiment and then replayed, and thus it is possible that right PAC activity in the non-driving part of the experiment may have occurred as the driver anticipated the trajectory of operation.

### Driver rehabilitation after brain injuries

The useful field of view of patients with right cerebral hemisphere injuries becomes significantly narrower than that of patients with left cerebral hemisphere injuries, and stroke survivors find it difficult to drive left curves after a stroke [46]. In addition, because changes in visuospatial cognition may result in a decline in driving skills, the resumption of driving after a stroke is more difficult for patients with lesions in the right hemisphere than for patients with lesions in the left hemisphere [46–48]. These previous reports are compatible with our results showing a greater response to road alignment in the right hemisphere than in the left. In helping patients with brain lesions to the bilateral PFC, the right PM, or the right FEF, it may be necessary to focus more on left curve driving.

However, the actual rate of accidents in left and right curves differ in different reports. More left curve accidents are reported by the Japan Safe Driving Center (1988) [49], the Institute for Traffic Accident Research and Data Analysis (2007) [50], Choi (2010) [51], and the National Highway Traffic Safety Administration (2008) [52]; and more right curve accidents are reported by the Society of Osaka Traffic Scientific Research (2000) [53], and the National Police Agency (2013) [54]. Differences in accident rates in left and right curves may be caused by multiple factors: for example, in addition to differences in difficulty in driving left and right curves and differences in brain activity, factors like traffic volume and time of driving may be involved.

### Application to intelligent transportation systems

In order to drive curves safely, a driver must be provided with information enabling him or her to understand the road alignment and maintain appropriate speeds. Conventional safety measures include lights and signs placed along or over the road. However, their effects on the driver have not been evaluated. In this study, PM activity, which is related to motion based on visual information [32], decreased in right curves and increased in left curves. It may be possible to evaluate the effects of lights and signs used for alerting drivers by measuring changes in brain activity during curve driving.

Accident rates are higher in curves than on straight sections when the curve radius is less than 1000 m. This tendency becomes more pronounced as the curve radius becomes less than 500 m [49]. It is thus possible that differences in the radius of curvature of the curve may also affect brain activity, and the question of what degree of curvature causes significant differences in brain activity between right and left curve driving is a potentially interesting subject for study. Measurement of brain activity using fNIRS may be able to provide suggestions for applying brain science to road design.

### Advantages and limitations of a vector-based approach

A vector-based approach has three advantages. First, brain activity can be evaluated using ***ΔO*** and ***ΔD*** simultaneously rather than a single index. ***ΔCOE*** and ***ΔCBV*** can then be calculated from ***ΔO*** and ***ΔD***; and the interrelationships between the indices ***ΔO***, ***ΔD***, ***ΔCBV*** and ***ΔCOE*** can be analyzed on the same vector plane. Changes in each component index can thus also be observed separately from the overall phase, which is derived from the interaction of the 4 indices. The use of a vector-based approach has produced the angle *k*, the scalar *L*, and *PRI* as new hemodynamic indices. The angle *k*, representing oxygen exchange efficiency, and the scalar *L*, representing the amount of change in hemoglobin, are new indices of change in strength of brain activity. *PRI*, on the other hand, is a synthetic indicator that includes the angle *k*, which determines the phase of the vector, and *L*, which contributes the scalar component of the vector. *PRI* is thus an index of brain activity that includes changes in both ***ΔCOE*** and ***ΔCBV***. Much previous research using fNIRS has evaluated increases in ***ΔO*** alone, or taken increased ***ΔO*** and decreased ***ΔD*** as a typical response indicating neural activity, ignoring other responses. A vector-based approach is a way to study brain activity without ignoring all the possible patterns of response that can be observed from measuring ***ΔO*** and ***ΔD***.

The second advantage of a vector-based approach is that brain activity can be evaluated quantitatively and thus classified into 8 different patterns using the concept of phase. Brain activity evaluation has traditionally been relative, depending on changes in signal amplitude. The ability to measure brain activity quantitatively has made it possible to improve measurement sensitivity, and in fact, it has become possible to detect multiple types of initial dips and distinguish small hemodynamic responses during a task at the short latency of 1.5 s [23]. In the field of brain-computer interface (BCI), in which signal classification is required, the use of the vector-based approach may be able to contribute to the currently proposed analysis of average values and signal slopes [55]. The vector-based approach can significantly reduce the delay in signal interpretation caused by the lag in hemodynamic response. Both fNIRS and EEG have attracted attention as being suitable for non-invasive and real-time BCI applications [56–58]. fNIRS, with the improved functional spatial and temporal resolution that the vector-based approach provides, or even without it, will likely be increasingly useful in noninvasive real-time BCI applications.

The third advantage is that vector-based analysis clarifies the relationship between oxygen saturation and the overall reaction pattern of ***ΔO*** and ***ΔD*** [27]. In this study, the points at which the scalar *L* peaked for the vectors from the 5 sites activated in the curve sections (right FEF, right PM, bilateral PFC, left PAC) were distributed in Phases 3 and 4, in which oxygen consumption occurs regardless of the baseline. Using *PRI*, we were able to detect differences between the left and right curves in reaction intensity at these five sites. The relationship between *PRI* and the strength of neural activity, however, requires further study.

Hong and Nguyen (2014) [59] have formulated equations for the relationship between any stimulus and hemodynamic response. It will be necessary to clarify in the same way the relationship between any stimuli or strength of oxygen metabolism and the eight phases, in order to further improve the utility of the vector-based approach and the index *PRI*.

### Limitations of driving simulator experiments

Changes in gravity are less in a driving simulator than in actual vehicle driving. In a simulator, visual information (e.g. optical flow) is the main sensory information that arises from changes in driving direction and vehicle speed, while somatosensory feedback is minimal. Therefore, in driving experiments using a simulator, what has been detected is mainly brain activity related to visual information associated with driving.

At least three points require further study. One is the effect of the lane travelled. In this study, the vehicles traveled in the left of 2 lanes, and forward visibility around left and right curves is different depending on the lane of travel. Verification of the experiment will be necessary with participants driving in the right lane. A second point is the possible effect of neck rotation in left and right curves. The curves driven in the present study had a radius of curvature of R = 600 m. This is a fairly gentle curve compared to those on roadways in general (for roads with speed limits of 50 km/h, the radius of curvature is generally ≥100 m) [60]. In fact, the maximum steering angle in this experiment was around 15°, and we therefore considered head rotation in the curves to be very small. As for the effects on blood flow due to head rotation, there have been reports that head rotation does not cause significant changes in cerebral perfusion or blood flow in the cervical spine [61], or in ΔOxyHb [62]. In fact, in the time-series waveforms of ΔOxyHb and ΔDeoxyHb shown in Fig 4a, there were no large baseline fluctuations between the curve sections and the straight sections, nor were there symmetrical large baseline fluctuations in the regions of the cerebral cortex. We thus considered the effect of head rotation to be small. However, it would be desirable to clarify the effects of head rotation with further study, possibly including keeping the head in a fixed position, or using motion capture to detect head movement.

A third point requiring further study is the effect of the dominant hand and gender. In this analysis, we did not intentionally control for handedness when recruiting participants. This was because hand dominance is cannot reflected in traffic safety measures or the issuance of driver’s license, and steering is done with both hands. In addition, the proportion of right and left handedness in this study was approximately the same as that of the population in general [63], and the results of this study can thus be considered to conform to actual conditions on the road. Right-handed subjects have been targeted in many basic functional brain imaging studies. But for the sake of developing traffic safety measures that do not depend on the dominant hand of the driver, continued research will be needed to detect brain activity that is common to drivers of either hand dominance. However, lateralization of the cerebral hemispheres according to the dominant hand has been reported [64], and attention to handedness may also be required when resuming driving after brain injury. In the present study, we compared the right-handed and left-handed drivers, and found differences in 3 regions, outside of the 5 regions where differences between left and right curves were detected. In 2 of these regions, the right-handed group showed more activation than the left-handed group, and in the other region, the left-handed group showed more activation than the right-handed group. We also compared male and female groups and found 2 sites with more activation in the female group than in the male group. There is a need to follow this up with more detailed study of the possible influence of differences in handedness and gender on brain activity during vehicle operation.

## Conclusions

Although we did not find that all the participants found it easier to drive curves in the same direction, we detected significant differences in brain activity during left and right curve driving. In left curves, significant activity occurred mainly in the frontal lobe, suggesting the possibility that left curve driving requires more attention and judgment. In right curve driving, the left PAC was activated. Measurement of brain activity during vehicle operation was useful in elucidating the mechanisms of attention while driving. Findings obtained from measuring brain activity during driving are also likely to be useful in considering improvements in road environment and driver support.
